# Effect of Low-Load and Low-Volume Squat Training Combined with Plyometrics During a Full Season on Physical Performance in Young Soccer Players

**DOI:** 10.3390/sports13100360

**Published:** 2025-10-11

**Authors:** Felipe Franco-Márquez, Carmen Serrano-Cañadillas, Juan Manuel Yáñez-García, Juan José González-Badillo, David Rodríguez-Rosell

**Affiliations:** 1 Physical Performance & Sports Research Center, Universidad Pablo de Olavide, 41013 Seville, Spain; fframar@gmail.com (F.F.-M.); carmenserranopf@gmail.com (C.S.-C.); jjgbadi@gmail.com (J.J.G.-B.); davidrodriguezrosell@gmail.com (D.R.-R.); 2 Department of Sport and Informatics, Universidad Pablo de Olavide, 41013 Seville, Spain; 3 Department of Communication and Education, Universidad Loyola Andalucía, 41704 Seville, Spain; 4 Investigation in Medicine and Sport Department, Research, Development and Innovation (R&D+i) Area, Sevilla Football Club, 41005 Sevilla, Spain

**Keywords:** soccer, full-season, plyometrics, low load, low volume, training, resistance training

## Abstract

The aim of this study was to analyze the effects of a 24-week low-load, low-volume resistance training (RT) program combined with plyometric exercises on the physical performance of U-15 male soccer players. Thirty-two young soccer players were divided into a strength training group (STG) and a control group (CG). The STG added two RT sessions per week—using moderate loads (45–60% 1RM) and a low number of repetitions per set—combined with plyometrics to their regular soccer training, while the CG continued with only the field soccer training. Performance assessments (a running sprint test, a countermovement jump, and a progressive loading test in a full squat exercise) were conducted before and after each of three 8-week periods. Significant ‘time × group’ interaction in favor of STG was observed for T20 (*p* < 0.05), CMJ (*p* < 0.001), and all variables (*p* < 0.001) assessed during the full squat exercise. Significant changes between groups were observed in T10 (Post 1 and Post 3, *p* < 0.05), CMJ (Post 1, Post 2, and Post 3, *p* < 0.05–0.001), and all strength variables (Post 1, Post 2, and Post 3, *p* < 0.05–0.001). The findings of this study suggest that a training program based on weightlifting with light loads for a few repetitions per set combined with jumps and sprint exercises, in addition to regular soccer training, induces greater and earlier improvements in strength and sport-related actions (jumping and sprinting), compared with only field soccer training. Coaches and strength-conditioning coaches should consider using RT with low loads and low volume and performing each repetition as fast as possible as an effective stimulus to improve physical performance in key match-determining actions efficiently.

## 1. Introduction

Soccer is an intermittent sport in which high-intensity actions, such as accelerations, decelerations, jumps, changes of direction, and high-speed running are crucial for successful performance [1,2]. These actions require high rates of force production from the lower limb muscles [3]. Indeed, performance in sprints, vertical jumps, and changes of direction have been strongly correlated with lower limb strength [4]. Therefore, implementing appropriate resistance training (RT) programs seems necessary to develop muscle strength and performance in sport-related actions in soccer players, but especially in young players, as it allows players to obtain long-term benefits for their athletic careers [5]. In this regard, resistance, plyometric, and sprint training have all been shown to improve strength, motor skill performance, and speed, while reducing injury risk and aiding rehabilitation in soccer players [6,7].

Both short- and long-term strength training, whether alone or combined with plyometric and sprint training, have been found to enhance maximal lower limb strength, jumping ability, and sprint performance in young male soccer players [8,9,10,11,12,13]. However, the confounding effect of maturation on training adaptations, which could complicate the distinction between training-induced and growth-related strength gains, could complicate the training process [5].

Several studies have observed that short-term RT combined with plyometric training using light loads and reduced volume in young soccer players leads to greater improvements in strength, jumping, and sprinting than those attributable to maturation alone [9,12]. Additionally, the training effects tend to be larger in younger players (U13 > U15 > U17) [12]. Similarly, other studies have found that long-term RT, in addition to regular soccer training, produces greater improvements in maximal strength, jumping capacity, and sprint times than those observed over 1–2 years of maturation only [13,14,15,16]. However, these studies were conducted using heavy loads and repetitions at or close to muscle failure. In contrast, one study [10] showed that 26 weeks of RT with moderate loads and low volume, combined with jumps and sprints, accelerated physical performance development in young soccer players. Notably, U16 and U18 players matched or even surpassed U21 players in all physical performance measures. A limitation of this study; however, was the absence of age-matched control groups.

Thus, there remains a gap in our understanding of whether long-term combined RT and plyometrics leads to continuous improvements over time that exceed those produced by maturation alone. Therefore, this study aimed to analyze the effects of adding an RT program, with low loads and low repetitions per set, combined with a reduced number of repetitions per set of jumping and sprinting exercises, to typical technical–tactical soccer training on lower limb strength, jumping ability, and sprint performance in post-pubertal (14–15 years) soccer players over 24 in-season weeks.

## 2. Materials and Methods

### 2.1. Experimental Approach to the Problem

**Figure 1:**
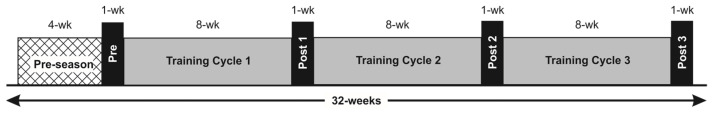
Schematic representation of study design.

A longitudinal and quasi-experimental study was used to analyze the effect of low-load and low-volume RT supplemented with plyometrics during a full season on strength, jumping capacity, and sprinting performance in young soccer players. For this, 40 young soccer players from the same club were randomly allocated into two groups: a strength training group (STG, *n* = 20) and a control group (CG, *n* = 20). The players who belonged to the STG performed free-weight squat training combined with plyometrics, whereas players assigned to the CG merely undertook typical soccer training. All participants were assessed before and after three 8-week training periods over a full season (32 weeks) using a sprint test of 20 m, a countermovement jump (CMJ) test, and a progressive loading test in the full squat (SQ) exercise. During the competition season, both groups performed 4 sessions of soccer training per week and played one official 90 min match. Each training session lasted about 1 h 40 min and comprised various skill activities at different intensities, small-sided games, and finally 10–20 min of continuous play or high-intensity interval training. The STG trained twice a week (Monday and Thursday) using the SQ exercise with moderate loads (45–60% 1RM) combined with jumps and sprint exercises. Between the initial tests (T1) and the final tests (T4), there were 28 weeks of training from the beginning of September to the end of April. During the 4 weeks preceding this study (pre-season period), 8 preliminary familiarization sessions (twice a week) were undertaken with the purpose of emphasizing proper execution technique in the full squat and CMJ. A schematic representation of the study design is presented in Figure 1.

### 2.2. Participants

Forty under-15 trained soccer players from the same academy were involved in this study. After the initial evaluation, players were matched according to their performance in the countermovement jump (CMJ) and then randomly assigned to either an STG or a CG. Only players who participated in at least 90% of all training sessions were included in the statistical analysis. Seven players were not taken into account because they were a) injured (n = 3), b) transferred to another club (n = 1), or c) absent from some of the evaluation sessions (n = 4). Thus, 32 of the 40 players who initially enrolled in the study were ultimately included in the statistical analysis: 17 players in the STG (age: 14.7 ± 0.5 years, height: 1.71 ± 0.05 m, weight: 60.3 ± 6.4 kg) and 15 players in the CG (age: 14.7 ± 0.5 years, height: 1.70 ± 0.05 m, weight: 64.3 ± 6.9 kg) remained for statistical analysis. For a study of these characteristics, using a repeated-measures ANOVA with a between (intervention: STG vs. CG) and a within factor (test: Pre vs. Post1 vs. Post2 vs. Post 3) and interactions, assuming an effect size of f = 0.25 (medium), α = 0.05, power (1–β) = 0.90, and a correlation among repeated measures = 0.60, the analysis indicated that a minimum of 26 participants (13 per group) would be required (G*Power software, version 3.1.9.7, University of Düsseldorf, Düsseldorf, Germany). Therefore, our sample meets the required criteria. All participants were soccer trained for more than 3 years and were injury free for at least 6 months before participating in this study. Subjects had no experience in strength training, and they did not perform RT or plyometric training as part of their normal training routine. Coaches and parents were informed about the different test procedures performed during the study. Parental/guardian consent for all players involved in this investigation was obtained. The study was conducted according to the Declaration of Helsinki and was approved by the Research Ethics Committee of Pablo de Olavide University (EH-1/2015).

### 2.3. Testing Procedures

All assessments were conducted in a single session. During the testing session, each soccer player performed a 20 m sprint test, a vertical jump test (CMJ), and a progressive loading test in the full squat (SQ) exercise, in that order. Before the physical performance assessment, all participants carried out a general standardized warm-up consisting of 5 min of jogging on the court, 3 sets of progressively faster 30 m running accelerations followed by 5 progressively higher-intensity jumps. Exactly the same testing protocol was carried out during all testing sessions. At least three experienced researchers supervised the testing to ensure correct and consistent techniques in all tests. Strong verbal encouragement was provided during all assessments to motivate participants to make a maximal effort.

#### 2.3.1. Running Sprint Test

Players carried out 2 maximal 20 m running sprints (3 min rest) on a synthetic indoor running track, and the best of both attempts was kept for analysis. The specific warm-up protocol consisted of one 0–40 m sprint at 80% effort, two 0–30 m sprints at 90% effort, and one 0–20 m sprint at maximal effort. Photocell timing gates (Witty wireless training timer, Microgate, Bolzano, Italy) were set up at a height of 1.10 m above ground level and were placed at 0, 10, and 20 m so that the times to cover 0–10 m (T10) and 0–20 m (T20) could be determined. A standing start, with the lead-off foot placed 1 m behind the first timing gate, was used. The coefficients of variation (CVs) for test–retest reliability for T10, T20, and T10–20 were 2.64%, 1.88, and 1.63%, respectively. The intraclass correlation coefficients (ICCs) were 0.83 [95% confidence interval (CI): 0.71–0.96] for T10, 0.92 (95% CI: 0.83–0.97) for T20, and 0.96 (95% CI: 0.91–0.99) for T10–20.

#### 2.3.2. Countermovement Jump Test

A CMJ was performed with the soccer players standing in an upright position on a contact platform (Optojump Next, Microgate, Bolzano, Italy) with their hands on their hips to avoid arm swings. From this position, participants performed a fast downward movement followed by a fast upward vertical movement as high as possible, all in one sequence, trying to reach the maximum possible height. Participants were instructed to land in an upright position and to bend their knees after landing. The subjects were required to take off and land within the same area to ensure the jump was completely vertical. The jump was not considered if the subject did not take off or land within the designated area. Five trials were completed with 45 s rest between each trial. The highest and lowest values were discarded, and the resulting mean value was kept for analysis. The specific warm-up consisted of 2 sets of 10 repetitions of the squat exercise without extra load (2 min rest), 5 CMJs at progressive intensity (20 s rest), and 3 maximal CMJs (30 s rest). The CV was 2.13% and the ICC was 0.99 (95% CI: 0.99–1.00).

#### 2.3.3. Progressive Loading Test in the SQ Exercise

A progressive loading test was performed in the full SQ exercise on a Smith machine (Multipower Fitness Line, Peroga, Murcia, Spain). The players performed the SQ from an upright position, descending (eccentric phase) in a continuous motion until the posterior thighs and calves made contact with each other, then immediately reversed the motion and ascended back to the starting position. The eccentric phase was performed at a controlled velocity (~0.50–0.60 m·s^−1^), whereas players were required to always execute the concentric phase at maximal intended velocity in all repetitions. The specific warm-up consisted of 2 sets of 6 repetitions (2 min rests) with 10 kg. The initial load was set at 20 kg for all players and was gradually increased in 10 kg increments until the mean propulsive velocity (MPV) was lower than ~1.00 m·s^−1^, which corresponds to ~60% 1RM (1). The players performed 3 repetitions with each load. The interset recovery time was 3 min. The exact same warm-up and progression of absolute loads were repeated in all testing sessions for each participant. A linear velocity transducer (T-Force System, Ergotech, Murcia, Spain) was used to register bar velocity. Only the best repetition at each load, according to the criterion of fastest MPV, was considered for subsequent analysis. The following variables derived from this test were used for analysis: (i) The 1RM calculated for each individual from the MPV attained against the heaviest load (kg) lifted in the progressive loading test, as follows: (100 × load)/(–5.961 × MPV2) − (50.71 × MPV) + 117 (1); (ii) average MPV attained against all absolute loads common to Pre and Post tests (AV); and (iii) MPV attained against 20 kg (MPV20), 30 kg (MPV30), 40 kg (MPV40), and 50 kg (MPV50).

### 2.4. Strength Training Program

In addition to their typical soccer training, players in the STG conducted two specific strength training sessions, each lasting 30–40 min, before field training. During the season, three cycles of 8-week strength training were performed with 1 week of recovery between each cycle. The week of recovery was used to carry out the corresponding tests to assess the physical condition of the players. Strength training was carried out, including essential exercises such as the SQ, unloaded jumps, and sprint exercises. Table 1 shows in detail the exercises, number of sets, repetitions, and the exercise intensities. Exercises were performed in the same order in which they appear in each table (e.g., in session 2, training exercises were performed in the following order: SQ, CMJ, SPTJ, and sprint), and all training sets of an exercise had to be completed before performing the following training exercise. The loads used by each player in the SQ exercise were assigned according to 1RMest obtained in the tests of progressive loads in the SQ exercise. The relative loads used in the SQ exercise during all training cycles ranged from 45 to 60% 1RM. Thus, during the season, the range of relative load for this exercise remained constant and the only change was the absolute load, which was adjusted after each test period. In each strength training session, the SQ exercise was combined with a low volume of plyometrics training. Approximately 2–3 min rest periods were allowed between each set and each exercise. The players were instructed to perform all exercises at maximal intended velocity [17]. Two trained researchers supervised each workout session and recorded the compliance and individual workout data during each training session. All sessions were carried out under the same environmental conditions (~20 °C and ~60% humidity) in a gym attached to the soccer field where the typical technical–tactical sessions were performed. During all training sessions, players carried out a general standardized warm-up which consisted of five minutes of jogging on the court, 3 progressively higher-intensity 10 m accelerations, 3 progressively higher-intensity 20 m accelerations, and finally 5 progressively higher-intensity CMJs.

### 2.5. Statistical Analysis

Standard statistical methods were used for the calculation of means and standard deviations (SD). The normality of distribution of the variables at Pre was examined with the Shapiro–Wilk test, and the homogeneity of variance across groups (STG vs. CG) was verified using Levene’s test. A one-way random effects model (model 2.1) ICC was used to determine relative reliability with a 95% confidence interval. Absolute reliability was reported using the CV. A 2 (group: STG vs. CG) × 4 (time: Pre vs. Post 1 vs. Post 2 vs. Post 3) factorial ANOVA with Bonferroni’s adjustment was used to analyze the differences between groups. Beyond the null hypothesis testing, we also evaluated the clinical relevance of the findings by considering the magnitude of change [18]. Effect sizes (ESs) were estimated using Hedges’ g [19]. The probabilities of beneficial or harmful effects were then expressed qualitatively: <1% (almost certainly not), 1–5% (very unlikely), 5–25% (unlikely), 25–75% (possible), 75–95% (likely), 95–99% (very likely), and ≥99% (almost certain). When both beneficial and harmful probabilities exceeded 5%, the outcome was deemed unclear. Statistical significance was accepted at *p* < 0.05. The null hypothesis tests were performed using SPSS software version 26.0 (SPSS, Chicago, IL, USA).

## 3. Results

**Figure 2:**
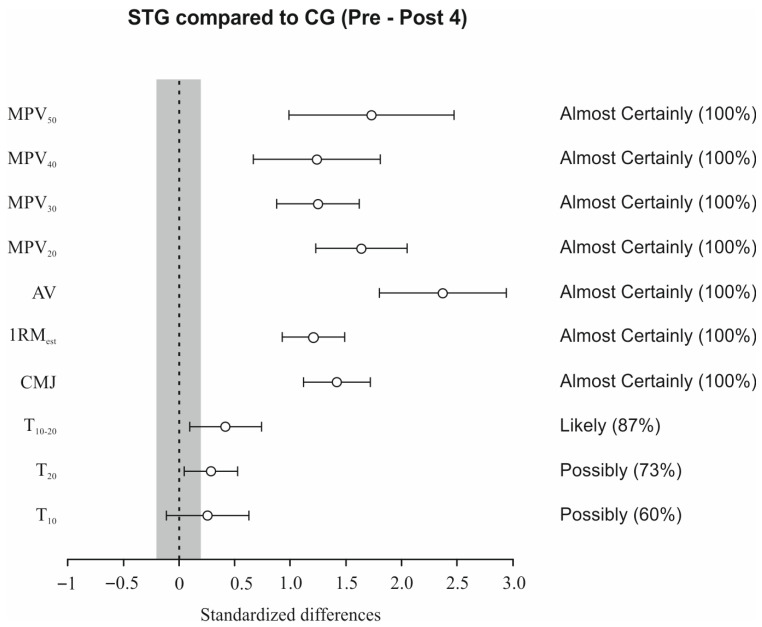
Pre to Post 3 changes (90% CI) in sprint times over 10 m (T10), 10–20 m (T10–20), and 20 m (T20), countermovement jump (CMJ), estimated one-repetition maximum (1RMest), mean velocity across absolute loads (AV), and bar velocity at 20, 30, 40, and 50 kg (MPV20–50) for STG compared with CG. Shaded regions denote trivial effects. Probabilities of practically meaningful differences favoring STG are reported in brackets. Note: The grey area indicates the minimum detectable change.

**Figure 3:**
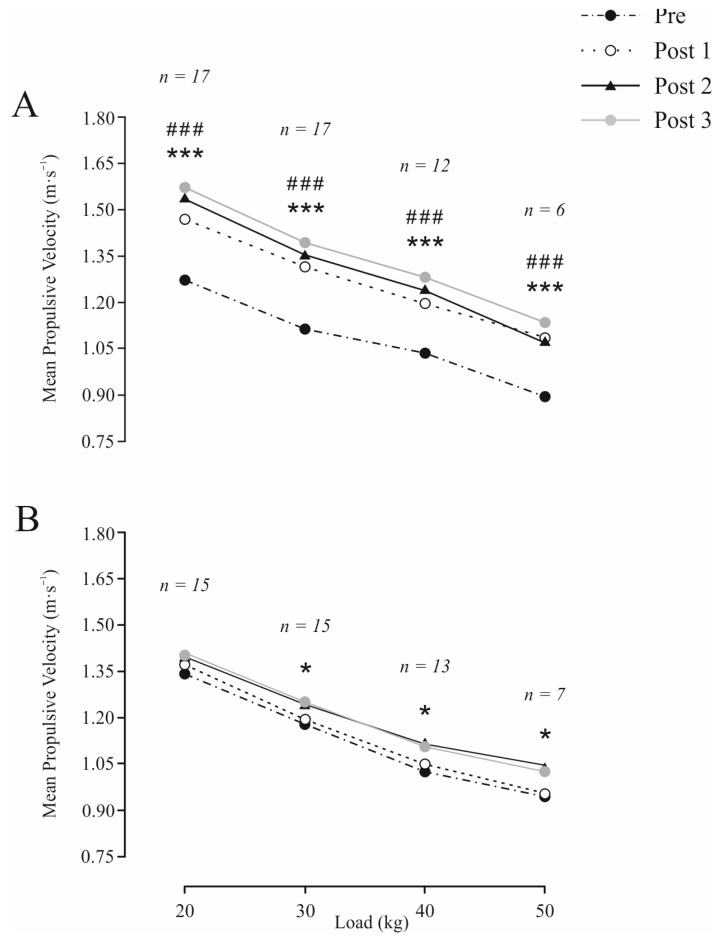
Load–velocity curve in full squat exercise obtained in strength training group (**A**) and control group (**B**) before (Pre) and after 6-week (Post 1), 12-week (Post 2) and 18-week (Post 3) training period. Values are means ± SD. Significant difference within group: * *p* < 0.05; *** *p* < 0.001. Significant interaction ‘time × group’: ### *p* < 0.001. Note: The reduction in sample size across successive loads occurred because certain participants were not required to progress to those loads during the initial isoinertial squat loading test.

Data for all variables analyzed were homogeneous and normally distributed. There were no significant differences between groups (STG vs. CG) at baseline in any of the variables studied. Significant ‘time × group’ interaction in favor of STG was observed for T20 (*p* < 0.05), CMJ (*p* < 0.001) and all variables (*p* < 0.001) assessed during the SQ exercise (Table 2). Significant changes between groups were observed in T10 (Post 1 and Post 3, *p* < 0.05), CMJ (Post 1, Post 2, and Post 3, *p* < 0.05–0.001), and all strength variables (Post 1, Post 2, and Post 3, *p* < 0.05–0.001) (Table 2 and Figure 2). In addition, STG presented an Almost Certainly greater effect on CMJ and strength variables, and a Likely (T10–20) and Possibly (T10 and T20) greater effect on sprint performance than CG (Figure 3). STG showed significant improvements from Pre to Post 1, Post 2, and Post 3 in all variables assessed, except in T10 and T10–20, which only showed significant changes from Pre to Post 3. The CG resulted in significant changes from Pre in 1RMest (Post 2 and Post 3), and MPV attained against 30 kg (Post 3), 40, and 50 kg (Post 2 and Post 3). Greater intragroup ESs were found for STG than for CG in all variables (Table 2 and Figure 2).

## 4. Discussion

The findings of this study indicate that implementing a SQ training program with light loads and low volume, in combination with jumping and sprinting exercises, over 24 weeks leads to significant and relevant improvements in strength, jumping, and sprinting performance in young male soccer players compared to a CG. These results suggest that strength training programs characterized by low fatigue levels yield superior and more rapid gains in physical performance than those attributable to natural maturation alone, as the CG showed no substantial improvements across all tests (Table 2).

Several studies have analyzed the effect of applied RT programs either alone or combined with jumping and sprinting exercises in young soccer players. However, most of them have focused on analyzing the short-term (6–8 weeks) effect [8,9,12,20,21], while few have addressed the long-term effect [11,14,16,17]. Considering the changes observed after the first training cycle (8 weeks), our results are consistent with previous studies of similar duration and training characteristics [10,13,15,16]. However, other studies that implemented strength training did not find significant changes in CMJ or sprinting performance [11,20,21]. The discrepancies in our results could be due to the high level of fatigue induced by using heavy squat training combined with repetitions to failure (4–6 RM) [21], the high volume (or total absence) of plyometric exercises [11], or concurrence with a high volume of endurance training [20], which could limit the positive adaptations produced by strength training in this population.

Regarding the long-term effects, the magnitude of changes (%) for the STG in the main variables measured (except for the 1RMest) was greater (CMJ = 19.8%; T20 = −1.6%; 1RM = 41.8%) than those reported in a previous study (CMJ = 5.7%; T30 = −0.22%; 1RM = 90.3%) applying comparable training intervention over a full season in young soccer players [16]. The gains found in the present study were even similar to those presented by Keiner et al. [15], despite their study conducting an RT program for a longer period (two full seasons) in soccer players of similar age. In both previous studies [15,16], RT programs were configured using heavy loads (>70–80% 1RM) and training volumes near or at muscle failure in each training set, and were not combined with plyometric exercises, which could compromise adaptations to high-speed actions (i.e., jumps and accelerations) [9,10,12,17,22,23]. In addition, it is worth noting that the initial level (relative strength: 1RM/body mass) of the soccer players in previous studies [15,16] was lower (relative strength: 0.40–0.70) than those presented by the soccer players in our research (relative strength: 1.06), which could lead to further gains in strength performance [12,23,24,25]. Therefore, according to previous studies [9,10,12,17,22,26], our results suggest that RT programs with light loads and low volume combined with a reduced volume of plyometrics are equally effective in improving physical performance in young soccer players to RT involving sets to muscle failure, with the mitigating factor that it generates a lower degree of fatigue during each training session (i.e., less technical–tactical deterioration during the subsequent soccer field session), requires less training time, recovery time, and even fewer material resources.

On the other hand, Keiner et al. [14] also found lower improvements in both “plyometric” and “strength” training groups (CMJ = 2.7–8.3%; COD = −0.3–6.2%; 1RM = −0.58–11.8%) after 10 months of intervention. As mentioned before, the difference in our results could be due to the non-combination of the two types of training (RT and plyometrics), which is more effective for inducing gains in sports-related actions [27,28]. In addition, the difference in the chronological ages of the soccer players (U-19 vs. U-15) could be a relevant factor to consider when analyzing the differences in performance gains, since it is well known that biological maturation plays a role in the magnitude of adaptation to strength training programs [10,12].

In contrast, Sander et al. [13] obtained greater gains in sprinting and strength performance after two full seasons using only heavy-load squat training (T20 = −4.3% and −5.4%, for U-15 and U-13, respectively). The differences between our results are mainly conditioned by the duration of the training period (one season vs. two seasons). However, it is necessary to assess the best procedure to obtain the greatest performance gains in young soccer players, because strength training, especially in the early stages, should be prescribed in terms of efficiency (performance gains relative to the degree of effort and time invested) rather than effectiveness.

The relative changes and effect sizes reported in the present investigation closely align with those found in González-Badillo et al. [10] following a 26-week training period (CMJ = 10.5%, ES = 0.91; T20 = −0.66%, ES = 0.23). This consistency may be attributed to the analogous training protocols implemented in both studies, characterized by a similar combination of exercises and training loads. Furthermore, the participants in both samples were of comparable chronological age categories (U-15 and U-16), thereby providing a plausible explanation for the positive trends observed in both interventions.

Therefore, the differences in the induced changes in strength, jumping, and sprinting performance following long-term strength training could be explained by several factors such as the initial strength level, the relative load and volume, the type and number of exercises, or even the method of measuring the 1RM. In any case, it appears that an adequate combination of low-fatigue and high-speed RT and plyometric exercises could produce greater and faster changes than isolated heavy-load RT, especially for sport-related actions (i.e., the RFD) over short- and long-term periods [9,10,12,17,22,23].

Finally, analyzing the evolution of training effects during the season, it was observed that the first training cycle resulted in greater performance adaptations compared to the following two cycles of the same duration. The vertical jump and strength variables showed progressive improvement throughout the season, although to a greater extent in the initial phase, while sprinting performance improved fundamentally in the first cycle and was then maintained until the end of the season. Based on these results, it could be deduced that the training stimulus provided was insufficient to induce improvements in sprinting. On the other hand, the interference of specific field training and the accumulated load during the matches could be a factor to consider when explaining these results. Thus, it would have been advisable to introduce exercises with a greater horizontal force application component, such as sled-resisted sprinting, to try to maximize performance gains in this type of activity.

### Limitations

This study has some limitations that should be acknowledged. First, the relatively small sample size could limit the statistical power of the analyses and the external validity of the findings to broader populations of young soccer players. Nevertheless, the homogeneity of the sample in terms of age, performance level, and training background strengthens the internal validity of the study and allows for a clearer interpretation of the training-induced adaptations and makes it possible to replicate this training program in other young male soccer players. However, it must also be acknowledged that there are additional factors which may potentially impact strength adaptations, and future research may consider the possibility of their inclusion as control variables (e.g., nutrition, body composition variables, rest patterns, specific field load). Second, the limited improvements observed in sprint performance may be explained by the training stimulus applied across the training cycles, which might have been insufficient to sustain continued adaptations beyond the initial phase of the intervention. In addition, the training design did not incorporate exercises with a stronger complementary horizontal force application component (e.g., resisted sprinting), which could have further enhanced sprint-specific gains. Third, the interference of concurrent soccer-specific training and the accumulated match load likely influenced the effectiveness of the strength program. Despite these limitations, the present study provides valuable evidence on the long-term effects of a low-load, low-volume RT program combined with plyometrics, highlighting its potential as an efficient and effective strategy to improve strength and performance in sport-related actions in young soccer players.

## 5. Conclusions

The main findings of this research showed that an RT program based on weightlifting with light loads and few repetitions per set combined with jumps and sprinting exercises, in addition to regular field soccer training, induced significant and early changes in physical performance in young soccer players compared with only field soccer training.

Thus, applying strength training with these characteristics is not only an effective conditioning method for training but also an efficient process for the enhancement of sport performance in young soccer players, specifically for the determinant actions of the match. However, the total load of horizontal acceleration stimulus could be a limitation of adaptations in performance, especially for sprinting.

## 6. Practical Applications

The findings of this study enhance our understanding of how to monitor and dose training load in resistance exercises for young soccer players. Accordingly, two primary practical implications can be drawn from this investigation to inform coaches in optimizing the training process of young male soccer players. First, RT with low loads lifted as fast as possible combined with plyometrics is an effective stimulus to improve physical performance to a greater extent and more quickly without the need to train close to failure and create excessive fatigue. Second, considering the low volume of each training session, strength training with these characteristics could be easily integrated into the week (twice a week) before normal field soccer training.

## Figures and Tables

**Table 1:** Characteristics of strength training program in all training cycles.

Exercises	Sessions															
	1	2	3	4	5	6	7	8	9	10	11	12	13	14	15	16
**SQ (S** × **R)** **(%1RM)**	2 × 8	3 × 8	3 × 8	3 × 8	2 × 6	3 × 6	3 × 6	3 × 6	2 × 6	3 × 6	3 × 6	3 × 6	2 × 4	3 × 4	3 × 4	3 × 4
	(45%)	(45%)	(45%)	(45%)	(50%)	(50%)	(50%)	(50%)	(55%)	(55%)	(55%)	(55%)	(60%)	(60%)	(60%)	(60%)
**CMJ (S** × **R)**	3 × 5		3 × 5	2 × 4	3 × 5		3 × 5	2 × 4	3 × 4	3 × 4	3 × 4	3 × 4	3 × 5		3 × 5	
**SPTJ (S** × **J)**	3 × 8	3 × 8	4 × 8	4 × 8	3 × 10	3 × 10	4 × 10	4 × 10	3 × 12	3 × 12	4 × 12	4 × 12	3 × 14	3 × 14	4 × 14	4 × 14
**Sprint (R** × **D)**		3 × 20 m		4 × 20 m		3 × 20 m		4 × 20 m	3 × 20 m	3 × 20 m	3 × 20 m	3 × 20 m		4 × 20 m		4 × 20 m
**COD (R** × **T)**		3 × 10 s		3 × 10 s		3 × 10 s		3 × 10 s		4 × 10 s		4 × 10 s		4 × 10 s		4 × 10 s

SQ: full squat exercise; CMJ: countermovement jump; 1RMest: estimated one-repetition maximum; SPTJ: step phase triple jump; COD: changes of direction; S × R: sets × repetitions; R × D: repetitions × distance; S × J: sets × number of jumps; R × T: repetitions × duration.

**Table 2:** Changes in selected physical performance variables during all seasons in each group (STG and CG).

	Pre	Post 1			Post 2			Post 3		
Variable	Mean ± SD	Mean ± SD	Δ (%)	ES (CI90%)	Mean ± SD	Δ (%)	ES (CI90%)	Mean ± SD	Δ (%)	ES (CI90%)
**T10**										
**STG**	1.77 ± 0.06	1.74 ± 0.06 †	−1.8	−0.48 (0.33)	1.74 ± 0.06	−1.3	−0.34 (0.24)	1.73 ± 0.06 *†	−2.3	−0.53 (0.23)
**CG**	1.79 ± 0.07	1.79 ± 0.06	−0.3	−0.07 (0.33)	1.78 ± 0.05	−0.9	−0.22 (0.33)	1.78 ± 0.05	−0.8	−0.19 (0.29)
**T20 ‡**										
**STG**	3.10 ± 0.11	3.06 ± 0.10 *	−1.3	−0.34 (0.18)	3.06 ± 0.12 *	−1.3	−0.27 (0.13)	3.05 ± 0.11 ***	−1.6	−0.43 (0.13)
**CG**	3.12 ± 0.10	3.13 ± 0.09	0.2	0.07 (0.27)	3.10 ± 0.09	−0.6	−0.18 (0.24)	3.10 ± 0.08	−0.6	−0.17 (0.22)
**T10–20**										
**STG**	1.33 ± 0.07	1.31 ± 0.06	−1.2	−0.24 (0.14)	1.31 ± 0.06	−1.6	−0.30 (0.16)	1.30 ± 0.06 **	−2.1	−0.39 (0.18)
**CG**	1.32 ± 0.04	1.32 ± 0.05	0.5	0.15 (0.35)	1.31 ± 0.04	−0.2	−0.05 (0.40)	1.31 ± 0.04	−0.2	−0.05 (0.32)
**CMJ ‡‡‡**										
**STG**	33.2 ± 4.7	36.5 ± 5.6 ***†	9.7	0.61 (0.18)	38.3 ± 5.3 ***††	15.2	0.93 (0.19)	39.8 ± 5.8 ***†††	19.8	1.19 (0.19)
**CG**	32.9 ± 2.3	33.2 ± 2.8	0.9	0.12 (0.20)	33.8 ± 2.9	2.6	0.34 (0.28)	33.3 ± 2.5	1.0	0.14 (0.32)
**1RM_est_ ‡‡‡**										
**STG**	64.1 ± 11.7	81.4 ± 14.6 ***	26.9	1.16 (0.20)	84.2 ± 12.5 ***	32.0	1.35 (0.22)	90.2 ± 11.8 ***†	41.8	1.70 (0.25)
**CG**	72.0 ± 14.8	73.0 ± 13.2	1.8	0.08 (0.18)	76.0 ± 12.7 *	5.6	0.28 (0.33)	78.2 ± 13.3 **	9.3	0.41 (0.16)
**AV ‡‡‡**										
**STG**	1.11 ± 0.08	1.30 ± 0.11 ***†††	17.6	2.09 (0.38)	1.35 ± 0.07 ***†††	21.8	2.55 (0.36)	1.39 ± 0.09 ***†††	25.3	2.92 (0.45)
**CG**	1.15 ± 0.06	1.18 ± 0.06	2.4	0.42 (0.34)	1.23 ± 0.09	6.4	1.11 (0.74)	1.22 ± 0.07	6.0	1.05 (0.39)
**MPV_20_ ‡‡‡**										
**STG**	1.27 ± 0.14	1.47 ± 0.14 ***†	15.6	1.25 (0.29)	1.53 ± 0.11 ***††	21.0	1.65 (0.27)	1.57 ± 0.13 ***†††	23.9	1.85 (0.30)
**CG**	1.34 ± 0.10	1.37 ± 0.07	2.4	0.29 (0.30)	1.40 ± 0.14	4.0	0.48 (0.55)	1.40 ± 0.10	4.5	0.55 (0.33)
**MPV_30_ ‡‡‡**										
**STG**	1.11 ± 0.15	1.32 ± 0.15 ***†	18.7	1.18 (0.23)	1.35 ± 0.12 ***†	21.7	1.35 (0.24)	1.39 ± 0.12 ***††	24.9	1.53 (0.29)
**CG**	1.18 ± 0.12	1.19 ± 0.11	1.5	0.13 (0.13)	1.25 ± 0.12	6.1	0.54 (0.42)	1.25 ± 0.11 *	6.0	0.52 (0.25)
**MPV_40_ ‡‡‡**										
**STG**	1.04 ± 0.09	1.20 ± 0.10 ***††	15.5	1.62 (0.37)	1.24 ± 0.04 ***††	19.9	1.86 (0.37)	1.27 ± 0.07 ***†††	23.1	1.97 (0.56)
**CG**	1.03 ± 0.11	1.05 ± 0.11	2.4	0.20 (0.28)	1.12 ± 0.13 **	8.9	0.74 (0.43)	1.11 ± 0.10 *	8.1	0.68 (0.27)
**MPV_50_ ‡‡‡**										
**STG**	0.90 ± 0.05	1.09 ± 0.09 **†	21.0	3.19 (1.19)	1.07 ± 0.05 ***	19.6	2.98 (0.35)	1.14 ± 0.07 ***	26.7	3.96 (0.80)
**CG**	0.94 ± 0.09	0.95 ± 0.13	−0.2	−0.02 (0.55)	1.05 ± 0.09 **	10.9	1.02 (0.38)	1.03 ± 0.11 *	8.9	0.84 (0.51)

SD: standard deviation; Δ: percentage of change; ES: effect size; CI: confidence interval; STG: strength training group; CG: control group; T10: sprint time in 10 m; T20: sprint time in 20 m; T10–20: sprint time elapsed between 10 and 20 m; CMJ: countermovement jump; 1RMest: estimated one-repetition maximum in the full squat exercise; AV: average mean propulsive velocity attained against all absolute loads common to Pre- and Post-tests; MPV: mean propulsive velocity. Statistically significant “time × group” interaction: ‡ *p* < 0.05, ‡‡‡ *p* < 0.001. between-groups significant differences: † *p* < 0.05, †† *p* < 0.01, ††† *p* < 0.001. intra-groups significant differences from Pre: * *p* < 0.05, ** *p* < 0.01, *** *p* < 0.001.

## Data Availability

The data that support the findings of this study are available from the corresponding author upon reasonable request. The data are not publicly available due to privacy and ethical restrictions.
